# Pollen Food Allergy Syndrome in Allergic March

**DOI:** 10.3390/nu14132658

**Published:** 2022-06-27

**Authors:** Hiroki Yasudo, Kiwako Yamamoto-Hanada, Limin Yang, Mayako Saito-Abe, Miori Sato, Yumiko Miyaji, Mami Shimada, Seiko Hirai, Kenji Toyokuni, Fumi Ishikawa, Yusuke Inuzuka, Shigenori Kabashima, Tatsuki Fukuie, Yukihiro Ohya

**Affiliations:** Allergy Center, National Center for Child Health and Development, Tokyo 157-8535, Japan; yo-r@ncchd.go.jp (L.Y.); saito-myk@ncchd.go.jp (M.S.-A.); sato-m@ncchd.go.jp (M.S.); miyaji-y@ncchd.go.jp (Y.M.); shimada-ma@ncchd.go.jp (M.S.); hirai-s@ncchd.go.jp (S.H.); toyokuni-k@ncchd.go.jp (K.T.); ishikawa-f@ncchd.go.jp (F.I.); yusuke-inuzuka@hotmail.co.jp (Y.I.); kabashima-s@ncchd.go.jp (S.K.); fukuie-t@ncchd.go.jp (T.F.); ohya-y@ncchd.go.jp (Y.O.)

**Keywords:** allergic (atopic) march, Bet v 1, Cry j 1, pollen food allergy syndrome, sensitization

## Abstract

The association between pollen food allergy syndrome (PFAS) and allergic march remains unclear. In this prospective cohort study of the general population in Tokyo (T-Child Study), we found that sensitization to Cry j 1 and Fel d 1 at ages 5 and 9 years was associated with an increased risk of PFAS at 13 years old (at 5 years, Cry j 1: adjusted odds ratio aOR, 2.74; 95% confidence interval CI, 1.53–4.91; Fel d 1: aOR, 2.61; 95% CI, 1.31–5.19; at 9 years, Cry j 1: adjusted odds ratio aOR, 4.28; 95% confidence interval CI, 1.98–9.25; Fel d 1: aOR, 2.40; 95% CI, 1.33–4.32). In particular, sensitization to Bet v 1 at ages 5 and 9 years was associated with a strong risk of PFAS at the age of 13 years (at 5 years: aOR, 10.6; 95% CI, 2.64–42.5; at 9 years: aOR, 9.1; 95% CI, 4.71–17.6). PFAS risk by age 13 years was increased by any allergic symptom at 5 or 9 years, a combination of wheezing, eczema, and rhinitis, and Bet v 1 sensitization. Our findings suggest that PFAS may be associated with allergic march.

## 1. Introduction

Pollen food allergy syndrome (PFAS) is characterized by oral allergy syndrome (OAS), which is an immediate allergic symptom in the oral mucosa seen in patients with pollen allergy after eating fruits and vegetables [1,2]. PFAS is associated with plant-related allergen components found in fruits and vegetables, including lipid transfer proteins, profilin, and PR-10 proteins, and is caused by cross-reactivity between pollen allergens and fruit and/or vegetable allergens [3,4]. Birch pollen is a representative inhaled pollen that induces PFAS. In Europe, about half of the patients sensitized to birch pollen developed symptoms of PFAS after consuming fresh fruits and vegetables [5,6]. Moreover, a recent Japanese epidemiological study showed that the positivity rate of birch pollen-specific immunoglobulin E (IgE) was significantly higher in participants with PFAS symptoms than in those without these symptoms, suggesting that Bet v 1 sensitization could be associated with the development of PFAS [7].

The prevalence of PFAS may vary depending on the geographical differences in the trees, grass, and weeds that cause pollen allergy [3]. A cross-sectional study using prospective birth cohort data in Japan reported that the prevalence of PFAS in children aged 13 years was 11.7% [8]. Among them, 72.7% were sensitized to one or more of the tree-, grass-, and/or weed-derived allergens. However, the epidemiological studies on PFAS are all cross-sectional and/or retrospective, and the longitudinal studies on the association between allergen sensitization and the development of PFAS have yet to be conducted.

Allergic march refers to the natural history of allergic diseases that develop during infancy and childhood [9]. Classically, allergic march begins with atopic dermatitis (AD) and progresses to IgE-mediated food allergy (FA), allergic asthma (AA), and allergic rhinitis (AR). Allergic march is driven by both genetic and environmental factors, resulting in type 2 immune responses and sometimes causing elevated IgE levels [9,10]. Many epidemiological studies have suggested a link between AD, AA, and AR development [11,12,13]. According to the Canadian Healthy Infant Longitudinal Development birth cohort study, AD with sensitization to inhalants, foods, or both at 1 year of age increased the risk of physician-diagnosed asthma at 3 years of age, suggesting that food sensitization, possibly through transcutaneous sensitization, could be more likely to have additional atopic comorbidities [12]. Belgrave et al. [13] showed an increasing prevalence of AR and a decreasing prevalence of AD and wheeze with age using data from the Avon Longitudinal Study of Parents and Children and the Manchester Asthma and Allergy Study. However, no studies have evaluated the association between PFAS and AD, AA, and AR in terms of allergic march.

Thus, in this study, we aimed to evaluate the factors associated with the development of PFAS using a birth cohort study of the general population in the Tokyo Children’s Health, Illness, and Development Study (T-Child Study) [14,15,16,17,18].

## 2. Materials and Methods

### 2.1. Study Design, Setting, and Participants

**Figure 1:**
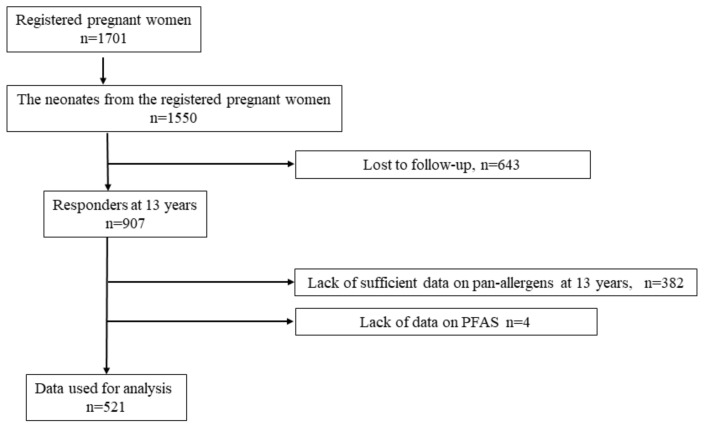
Flow chart of the participants included in this study. PFAS, pollen food allergy syndrome.

We performed a longitudinal study on the association between PFAS incidence in participants aged 13 years with allergic symptoms and/or IgE component sensitization in those aged 5–9 years using a prospective birth cohort study of the general population in the T-Child Study (Figure 1). The T-Child Study was conducted at the National Center for Child Health and Development (NCCHD) of the National Children’s Hospital in Tokyo, Japan.

A total of 1701 pregnant women who attended their first antenatal visit at the NCCHD were registered between 2003 and 2005. A total of 1550 newborn babies were enrolled in the study between March 2004 and August 2006. The target population in this study was children who were followed up until they reached the age of 13 years, completed all variables for questionnaires, and underwent medical examinations and blood tests.

### 2.2. Exposure Variables

The exposures evaluated in this study included wheezing, eczema, and rhinitis status and sensitization to the pan-allergens or indoor inhalant allergens listed in Table S1 at ages 5 and 9 years; wheeze status at ages 5 and 9 years was judged on the basis of the following questionnaire item at ages 5 and 9 years: “Has your child ever had wheezing or whistling in the past 12 months?”. Eczema status at age 5 or 9 years was judged on the basis of the following questionnaire item at age 5 or 9 years: “Has your child ever had itchy eczema in the past 12 months?”. Rhinitis status at ages 5 and 9 years was judged on the basis of the following questionnaire item at ages 5 and 9 years: “In the past 12 months, has your child had a problem with sneezing, or a runny, or blocked nose when he/she did not have a cold or the flu?”. A specific IgE of ≥0.3 ISU (ISAC standardized units) for the analyzed allergen was considered positive sensitization.

### 2.3. Outcome Variables

The outcomes evaluated in this study included hay fever, OAS, pollen allergy, and PFAS (Table S1). Hay fever was judged on the basis of the following questionnaire item at age 13 years: “Has your child ever had hay fever? (Hay fever ever at 13 y)”, and OAS was judged on the basis of the following questionnaire items at age 13 years: “Has your child ever had an itchy mouth or redness around his/her mouth after eating fruits and vegetables? (OAS ever at 13 y)”. Pollen allergy was evaluated on the basis of the definition of meeting both of the following criteria “Hay fever ever at 13 y” and “sensitization to pan allergens.” PFAS was evaluated on the basis of the definition of meeting both of the following criteria: “Pollen allergy ever at 13 y” and “OAS ever at 13 y”. The questionnaires were developed by certified allergists and epidemiologists. Component-specific IgE (sIgE) levels were analyzed using ImmunoCAP ISAC [19,20]. Pan-allergens are proteins that take part in key processes of organisms with highly conserved sequences and structures and belong to various families, such as profilin, polcalcin, non-specific lipid transfer protein (LTP), and pathogenesis-related protein family 10 (PR-10) [21]. Thirty-six pan-allergens derived from 20 plants were included in this study (Table S2).

### 2.4. Questionnaire Survey

A paper-based questionnaire in Japanese, which included the International Study of Asthma and Allergies in Childhood (ISAAC) [22,23,24] and clinical history of hay fever and OAS, was completed by the participants’ parents. The responses were used to evaluate the clinical symptoms of PFAS and other allergic diseases at ages 5, 9, and 13 years.

### 2.5. Blood Sampling and IgE Component Measurement

Serum samples were obtained from participants aged 5, 9, and 13. Allergen component sIgE antibody titers were measured using the commercially available multiplex array ImmunoCAP ISAC [19,20] (Thermo Fisher Scientific, Tokyo, Japan). The specimens were managed by an independent contract laboratory (SRL, Inc., Tokyo, Japan).

### 2.6. Bias and Study Size

To minimize sampling bias, all participants aged 13 years who completed the questionnaire surveys, underwent a medical check-up, and provided a blood sample for the T-Child Study were included. The 13-year-olds who participated in the study had been followed prospectively since their birth. The study size was not determined, as this study was an exploratory study.

### 2.7. Statistical Analysis

Descriptive statistics were used to analyze all outcomes. Potential confounders were familial history of allergic diseases, including rhinitis, smoking, pet ownership, sex, siblings, and family income. Univariate and multivariate logistic regression analyses were used to evaluate the association between allergic diseases, sensitization status at ages 5 or 9 years, and PFAS at the age of 13 years. These potential confounders were included in multivariate models to obtain adjusted odds ratios (aORs). Multiple imputation (MI) was used to manage missing values. We generated 20 data sets with the missing data imputed in MI processes. To show the robustness of the results from MI, the aORs based on the complete dataset (any with missing data were deleted) were presented as well. All associations based on these models are presented with ORs and 95% confidence intervals (CIs).

All statistical analyses were performed using R statistical software, version 4.0.3 (the R Foundation for Statistical Computing, Vienna, Austria).

## 3. Results

### 3.1. Participant Characteristics

Among the enrolled 1550 newborn babies, 907 participants were followed up and responded to the questionnaire at the age of 13 years. Some participants were excluded due to insufficient data on pan-allergens (n = 382) and PFAS (n = 4) at the age of 13 years. Thus, 521 participants were included in the analysis (Fig 1). The baseline characteristics of the eligible participants were evaluated. Of the 521 participants, 292 (56%) had a history of hay fever; 82 (15.7%) had a history of OAS; 264 (50.7%) had a pollen allergy; and 59 (11.3%) had a history of PFAS. A comparison between the participants included and excluded in our study showed differences in the number of “wheeze current at 5 years old” (*p* = 0.048), “rhinitis current at 5 years old” (*p* = 0.033), “pet keeping at 6 months” (*p* = 0.03), “siblings” (*p* = 0.001), “maternal history of allergy” (*p* = 0.049), and “maternal history of rhinitis” (*p* = 0.006) (Table S3).

### 3.2. Association of Allergen Sensitization at 5 or 9 Years of Age with PFAS Outcomes at 13 Years of Age

Table 1 shows the association between pan-allergen or aeroallergen sensitization at 5 and 9 years of age and PFAS at 13 years of age in univariate, MI, or multivariate complete-case analyses. Sensitization to any pan-allergen at 5 years of age was associated with an increased risk of PFAS at age 13 years in the MI analysis (adjusted odds ratio (aOR]), 3.23; 95% CI, 1.58–6.62). Sensitization to Cry j 1 or Fel d 1 at the age of 5 years was associated with an increased risk of PFAS at 13 years of age (Cry j 1: aOR, 2.74; 95% CI, 1.53–4.91; Fel d 1: aOR, 2.61; 95% CI, 1.31–5.19), whereas that to Der p 1, Der f 1, or Can f 1 at the age of 5 years was not significantly associated with an increased risk of PFAS at 13 years of age (Der p 1: aOR, 1.66; 95% CI, 0.92–2.99; Der f 1: aOR, 1.71; 95% CI, 0.94–3.09; Can f 1: aOR, 1.49; 95% CI, 0.48–4.60).

Interestingly, the sensitization to Bet v 1 at the age of 5 years was associated with the strongest risk of PFAS at the age of 13 years (aOR, 10.6; 95% CI, 2.64–42.5).

The association between allergen sensitization at early ages and PFAS at the age of 13 years was similar to that between sensitization at 5 and 9 years of age, although sensitization to any pan-allergen or Cry j 1 at age 9 years was associated with an even stronger risk of PFAS at the age of 13 years (pan-allergen: aOR, 8.17; 95% CI, 2.80–23.9, Cry j 1: aOR, 4.28; 95% CI, 1.98–9.25). Similar trends were observed in the complete-case analysis.

### 3.3. Association between Symptoms Suggestive of Allergic Diseases at 5 Years of Age and PFAS Outcomes at 13 Years of Age

Table 2 shows the association between symptoms suggestive of allergic diseases with or without sensitization to pan-allergen, Bet v 1, or Cry j 1 at the age of 5 years and PFAS at the age of 13 years in univariate, MI, or multivariate complete-case analyses. Any symptoms of current wheezing, current eczema, or current rhinitis at the age of 5 years were associated with a risk of PFAS at the age of 13 years in the MI analysis (wheeze current: aOR, 2.03; 95% CI, 1.09–3.80; eczema current: aOR, 2.26; 95% CI, 1.27–4.03; rhinitis current: aOR, 2.26; 95% CI, 1.27–4.00). Comorbidity of allergic diseases at the age of 5 years significantly affected the development of PFAS at the age of 13 years (wheeze current and eczema current: aOR, 3.15; 95% CI, 1.41–7.06; wheeze current and rhinitis current: aOR, 2.57; 95% CI, 1.23–5.35; eczema current and rhinitis current: aOR, 2.66; 95% CI, 1.39–5.09). Triple symptoms at the age of 5 years also significantly affected the development of PFAS at 13 years of age (aOR, 3.47; 95% CI, 1.34–8.98). Current rhinitis with sensitization to any pan-allergen and the presence or absence of the other symptoms were associated with a strong risk of PFAS at 13 years of age (rhinitis current with pan-allergen sensitization: aOR, 4.07; 95% CI, 2.22–7.46; wheeze current and rhinitis current with pan-allergen sensitization: aOR, 3.47; 95% CI, 1.31–9.17; eczema current and rhinitis current with pan-allergen sensitization: aOR, 3.93; 95% CI, 1.91–8.10).

Interestingly, current rhinitis with Bet v 1 sensitization strongly affected the development of PFAS at the age of 13 years (aOR, 9.87; 95% CI, 2.24–43.6), although the association between rhinitis with Bet v 1 sensitization and the other symptoms was not evaluated due to an insufficient number of the analyzed participants. Current rhinitis with Cry j 1 sensitization was associated with PFAS at the age of 13 years (aOR, 2.96; 95% CI, 1.57–5.55). However, the association between rhinitis with Cry j 1 sensitization complicated with other allergic symptoms and PFAS at the age of 13 years was only observed in patients with eczema and rhinitis with Cry j 1 sensitization (aOR, 2.50; 95% CI, 1.13–5.54).

### 3.4. Association between Symptoms Suggestive of Allergic Diseases at 9 Years of Age and PFAS Outcomes at 13 Years of Age

Table 3 shows the association between symptoms suggestive of allergic diseases with or without sensitization to pan-allergen, Bet v 1, or Cry j 1 at the age of 9 years and PFAS at the age of 13 years in univariate, MI, or multivariate complete-case analyses.

The MI analysis showed that current wheezing, current eczema, or current rhinitis at the age of 9 years was associated with a risk of PFAS at the age of 13 years (wheeze current: aOR, 3.84; 95% CI, 1.91–7.73; eczema current: aOR, 2.02; 95% CI, 1.10–3.71; rhinitis current: aOR, 4.90; 95% CI, 2.31–10.4). Comorbidity of allergic diseases at the age of 9 years significantly affected the development of PFAS at 13 years of age (wheeze current and eczema current: aOR, 7.10; 95% CI, 2.41–20.9; wheeze current and rhinitis current: aOR, 4.50; 95% CI, 2.18–9.32; eczema current and rhinitis current: aOR, 2.66; 95% CI, 1.39–5.08). Notably, triple symptoms at the age of 9 years strongly affected the development of PFAS at the age of 13 years (aOR, 7.80; 95% CI, 2.38–25.6).

Rhinitis with sensitization to pan-allergen, Cry j 1, or Bet v 1 sensitization at the age of 9 years was associated with a risk of PFAS at age 13 years of age, irrespective of concomitant symptoms, such as current wheezing or eczema. In particular, rhinitis with Bet v 1 sensitization markedly affected the development of PFAS at the age of 13 years (rhinitis current with Bet v 1 sensitization: aOR, 11.3; 95% CI, 5.50–23.3). Furthermore, rhinitis with Bet v 1 sensitization concomitant with wheezing and/or eczema greatly affected the development of PFAS at 13 years of age in the complete-case analysis (rhinitis current with Bet v 1 sensitization and wheeze current: aOR, 36.5; 95% CI, 6.44–207; rhinitis current with Bet v 1 sensitization and eczema current: aOR, 6.97; 95% CI, 2.32–21.0, rhinitis current with Bet v 1 sensitization, wheeze current, and eczema current: aOR, 35.5; 95% CI, 3.55–356), although the association between previous development of rhinitis and Bet v 1 sensitization concomitant with wheezing with or without eczema was not evaluated due to insufficient participants in MI analysis.

## 4. Discussion

In this study, we evaluated the manifestation of allergic march associated with the development of PFAS in a birth cohort study of the general population in Tokyo. Sensitization to pan-allergens at 5 and 9 years of age was associated with an increased risk of PFAS at 13 years of age. Sensitization to Cry j 1 and Fel d 1, but not Der p 1, Der f 1, or Can f 1, at the ages of 5 and 9 years was associated with an increased risk of PFAS at 13 years of age. In particular, the sensitization to Bet v 1 at ages 5 and 9 years was associated with a strong risk of PFAS at the age of 13 years. Previous development of any allergic symptoms of wheezing, eczema, or rhinitis at 5 or 9 years of age was associated with a risk of PFAS at the age of 13 years. Moreover, the previous development of triple symptoms of wheezing, eczema, and rhinitis strongly affected the development of PFAS at the age of 13 years. Previous development of allergic symptoms with Bet v 1 sensitization most significantly affected the development of PFAS at the age of 13 years.

We previously reported that the prevalence of PFAS was 11.7% in patients at 13 years of age in Japan, which was higher than what we had anticipated [8]. Many of the PFAS patients (72.7%) were sensitized to one or more of the tree-, grass-, and/or weed-derived allergens. These findings suggest that PFAS is becoming more common in adolescents, and pollen sensitization may be associated with PFAS. In this study, we showed that sensitization to pan-allergens, Bet v 1, Cry j 1, or Fel d 1, but not Der p 1, Der f 1, or Can f 1, at pre-school and school age could increase the risk of PFAS development in adolescence. In particular, Bet v 1 sensitization at pre-school or school age was the strongest risk factor for PFAS development in early adolescence. Although these were all aeroallergens, sensitization to pollen-derived allergens was a crucial risk factor for the subsequent development of PFAS. In addition, it is interesting that sensitization to Fel d 1, but not to Can f 1, was positively associated with PFAS development. Some studies have shown that dog ownership, but not cat ownership, is negatively associated with the occurrence of allergic comorbidities [25,26]. This suggests that the sensitization of dog-derived allergens could have a preventive effect on subsequent PFAS development, although further detailed studies are required.

This study also showed that antecedent development of allergic symptoms of wheezing, eczema, and/or rhinitis at pre-school or school age was a risk factor for PFAS development in early adolescence. Multiple atopic comorbidities are highly associated with subsequent PFAS development, suggesting that PFAS is related to a late phase of atopic march progression. We propose that PFAS should be included as one of the updated versions of allergic march. Atopic comorbidities and/or pollen sensitization, including Bet v 1, are increasing in many countries [27,28]. Our previous study showed that the proportion of IgE sensitization to Bet v 1 (birch) was 2.2% at age 5 years and 13.9% at age 9 years, and pediatric participants were also highly sensitized to Cry j 1 (Japanese cedar) (32.8% at age 5 years and 57.8% at age 9 years) [14]. Given that previous development of allergic symptoms and/or the sensitization to pollen, especially Bet v 1 at pre-school and school age, was a risk factor for PFAS in adolescence as shown in our study, it is possible that PFAS prevalence could increase in the future.

Classically, allergic march begins with AD and progresses to IgE-mediated FA, AA, and AR [9,29]. Several studies have suggested that the mechanisms underlying the development of atopic comorbidities could involve an impaired cutaneous barrier, resulting in cutaneous sensitization, thereby leading to a systemic Th2-dominant immune response to cutaneous inflammation [9,11]. It would be interesting to evaluate the association between atopic comorbidities, such as AD and FA, at early ages and subsequent PFAS development; however, we did not evaluate this association in this study.

This study has some limitations. Information on hay fever and OAS was based on questionnaire responses given by the children’s parents and not a doctor’s diagnosis. These aspects could add a factor of variability to the results, despite evaluating PFAS on the basis of IgE component sensitization data. Second, this study was conducted in Tokyo, a metropolitan area; thus, the results cannot be generalized to the general pediatric population in Japan. As pollen sensitization varies in regions and/or countries, the results could vary in regions where pollen sensitization patterns are different. This warrants further investigation of regional differences in the association between the sensitization to Bet v 1 and/or the previous atopic comorbidities and subsequent PFAS development.

## 5. Conclusions

In conclusion, to our knowledge, this is the first longitudinal study to show that the preceding allergic features of allergy march were risk factors for the development of PFAS. We showed that the previous development of wheezing, eczema, or rhinitis at 5 or 9 years of age was associated with the risk of PFAS at 13 years of age using a birth cohort study of the general population in Tokyo. Moreover, the previous development of triple symptoms of wheezing, eczema, and rhinitis strongly affected the development of PFAS at the age of 13 years. Previous Bet v 1 sensitization remarkably affected the development of PFAS at the age of 13 years. These findings suggest that previous allergic comorbidities and/or Bet v 1 sensitization are positively associated with PFAS. Thus, our results propose that PFAS may be associated with allergic march progression.

## Figures and Tables

**Table 1:** Association between allergen sensitization at 5 or 9 years and PFAS at 13 years of age.

	OR	95% CI		*p*	aOR ^†^	95% CI		*p*	aOR ^‡^	95% CI		*p*
		Lower	Upper			Lower	Upper			Lower	Upper	
At 5 years												
Pan-allergen sensitization (278)	3.52	1.72	7.17	<0.001	3.23	1.58	6.62	0.0014	3.06	1.42	6.62	0.0044
Bet v 1 sensitization (9)	17.4	4.22	71.9	<0.001	10.6	2.64	42.5	0.0010	11.1	1.67	73.8	0.013
Cry j 1 sensitization (150)	2.88	1.62	5.11	<0.001	2.74	1.53	4.91	<0.001	2.59	1.34	5.01	0.0048
Der p 1 sensitization (181)	1.59	0.90	2.82	0.11	1.66	0.92	2.99	0.096	1.47	0.75	2.90	0.26
Der f 1 sensitization (194)	1.65	0.94	2.92	0.083	1.71	0.94	3.09	0.078	1.65	0.84	3.23	0.14
Can f 1 sensitization (28)	1.32	0.44	3.97	0.62	1.49	0.48	4.60	0.49	2.11	0.65	6.89	0.21
Fel d 1 sensitization (59)	2.92	1.48	5.79	0.0021	2.61	1.31	5.19	0.0066	2.83	1.28	6.25	0.010
At 9 years												
Pan-allergen sensitization (291)	12.8	3.9	41.7	<0.001	8.17	2.80	23.9	<0.001	11.7	3.50	39.0	<0.001
Bet v 1 sensitization (63)	9.64	5.13	18.1	<0.001	9.10	4.71	17.6	<0.001	8.65	4.05	18.5	<0.001
Cry j 1 sensitization (268)	5.15	2.38	11.2	<0.001	4.28	1.98	9.25	<0.001	5.35	2.27	12.6	<0.001
Der p 1 sensitization (236)	1.84	1.03	3.31	0.040	1.82	0.99	3.35	0.055	1.79	0.92	3.48	0.085
Der f 1 sensitization (250)	1.94	1.07	3.52	0.030	1.83	0.98	3.39	0.058	1.72	0.88	3.38	0.11
Can f 1 sensitization (43)	1.59	0.67	3.78	0.29	1.40	0.58	3.41	0.46	1.93	0.73	5.13	0.18
Fel d 1 sensitization (118)	2.78	1.55	4.99	<0.001	2.40	1.33	4.32	0.0037	2.47	1.27	4.81	0.0076

^†^ Multiple imputations; ^‡^ complete data; confounding factors included sex, pet-keeping, smoking exposure, siblings, family income, and parental history of rhinitis. (N) Categorized number; aOR, adjusted odds ratio; CI, confidence interval; OR, odds ratio; PFAS, pollen food allergy syndrome.

**Table 2:** Association of allergic diseases at 5 years with PFAS at 13 years of age.

	OR	95% CI		*p*	aOR ^†^	95% CI		*p*	aOR ^‡^	95% CI		*p*
		Lower	Upper			Lower	Upper			Lower	Upper	
At 5 years												
Wheeze current (98)	2.10	1.15	3.86	0.016	2.03	1.09	3.80	0.027	1.98	0.99	3.94	0.052
Eczema current (128)	2.30	1.31	4.04	0.0038	2.26	1.27	4.03	0.0056	1.77	0.93	3.38	0.082
Rhinitis current (198)	2.40	1.38	4.16	0.0019	2.26	1.27	4.00	0.0055	2.23	1.19	4.18	0.0124
Rhinitis current, pan-allergen sensitization (87)	4.34	2.41	7.83	<0.001	4.07	2.22	7.46	<0.001	4.66	2.32	9.34	<0.001
Rhinitis current, Bet v 1 sensitization (8)	14.0	3.25	60.3	<0.001	9.87	2.24	43.6	0.0027	6.1	0.79	46.4	0.083
Rhinitis current, Cry j 1 sensitization (78)	3.22	1.74	5.96	<0.001	2.96	1.57	5.55	<0.001	3.67	1.79	7.52	<0.001
Wheeze current, eczema current (37)	3.23	1.47	7.07	0.0034	3.15	1.41	7.06	0.0055	2.85	1.16	7.00	0.022
Wheeze current, rhinitis current (52)	2.64	1.29	5.37	0.0076	2.57	1.23	5.35	0.012	2.18	0.95	5.04	0.067
Wheeze current, rhinitis current, pan-allergen sensitization (25)	3.31	1.32	8.30	0.011	3.47	1.31	9.17	0.013	3.13	1.01	9.69	0.048
Wheeze current, rhinitis current, Bet v 1 sensitization (1)	-	-	-	-	-	-	-	-	-	-	-	-
Eczema current, rhinitis current, Cry j 1 sensitization (23)	2.28	0.81	6.38	0.118	2.37	0.80	6.99	0.12	2.51	0.74	8.51	0.14
Eczema current, rhinitis current (73)	2.88	1.53	5.40	0.0010	2.66	1.39	5.09	0.0032	2.47	1.19	5.13	0.0151
Eczema current, rhinitis current, pan-allergen sensitization (45)	4.26	2.11	8.61	<0.001	3.93	1.91	8.10	<0.001	3.73	1.61	8.69	0.0022
Eczema current, rhinitis current, Bet v 1 sensitization (6)	-	-	-	-	-	-	-	-	-	-	-	-
Eczema current, rhinitis current, Cry j 1 sensitization (41)	2.79	1.29	6.04	0.0093	2.50	1.13	5.54	0.024	2.84	1.16	6.96	0.023
Wheeze current, eczema current, rhinitis current (23)	3.69	1.45	9.37	0.0062	3.47	1.34	8.98	0.011	3.03	0.99	9.27	0.052
Wheeze current, eczema current, rhinitis current, pan-allergen sensitization (16)	5.02	1.75	14.4	0.0026	4.91	1.67	14.4	0.0040	4.15	1.12	15.4	0.033
Wheeze current, eczema current, rhinitis current, Bet v 1 sensitization (1)	-	-	-	-	-	-	-	-	-	-	-	-
Wheeze current, eczema current, rhinitis current, Cry j 1 sensitization (14)	3.22	0.98	10.6	0.055	3.08	0.91	10.4	0.071	3.14	0.75	13.2	0.118

^†^ Multiple imputations; ^‡^ complete data; confounding factors included sex, pet-keeping, smoking exposure, siblings, family income, and parental history of rhinitis. (N) Categorized number; aOR, adjusted odds ratio; CI, confidence interval; OR, odds ratio; PFAS, pollen food allergy syndrome.

**Table 3:** Association between allergic diseases at 9 years and PFAS at 13 years of age.

	OR	95% CI		*p*	aOR ^†^	95% CI		*p*	aOR ^‡^	95% CI		*p*
		Lower	Upper			Lower	Upper			Lower	Upper	
At 9 years												
Wheeze current (56)	3.47	1.78	6.76	<0.001	3.84	1.91	7.73	<0.001	3.64	1.65	8.02	0.0014
Eczema current (108)	1.97	1.09	3.56	0.025	2.02	1.10	3.71	0.024	2.55	1.32	4.91	0.0052
Rhinitis current (291)	5.00	2.40	10.4	<0.001	4.90	2.31	10.4	<0.001	3.96	1.83	8.59	<0.001
Rhinitis current, pan-allergen sensitization (201)	7.93	3.89	16.2	<0.001	6.68	3.29	13.6	<0.001	6.78	3.16	14.6	<0.001
Rhinitis current, Bet v 1 sensitization (45)	12.5	6.28	24.8	<0.001	11.3	5.50	23.3	<0.001	9.0	4.00	20.0	<0.001
Rhinitis current, Cry j 1 sensitization (192)	6.04	3.14	11.6	<0.001	5.30	2.75	10.2	<0.001	5.67	2.73	11.8	<0.001
Wheeze current, eczema current (16)	6.72	2.40	18.8	<0.001	7.10	2.41	20.9	<0.001	7.09	2.12	23.7	0.0015
Wheeze current, rhinitis current (46)	4.14	2.06	8.33	<0.001	4.50	2.18	9.32	<0.001	3.92	1.73	8.90	0.0011
Wheeze current, rhinitis current, pan-allergen sensitization (37)	3.96	1.84	8.54	<0.001	4.02	1.82	8.88	<0.001	3.96	1.63	9.63	0.0024
Wheeze current, rhinitis current, Bet v 1 sensitization (9)	31.9	6.44	158	<0.001	-	-	-	-	36.5	6.44	207	<0.001
Eczema current, rhinitis current, Cry j 1 sensitization (35)	3.11	1.38	7.02	0.0064	3.07	1.33	7.07	0.0087	3.36	1.35	8.36	0.0093
Eczema current, rhinitis current (77)	2.69	1.44	5.02	0.0020	2.66	1.39	5.08	0.0033	3.46	1.71	7.00	<0.001
Eczema current, rhinitis current, pan-allergen sensitization (56)	3.25	1.64	6.43	<0.001	2.92	1.44	5.94	0.0031	4.36	2.04	9.30	<0.001
Eczema current, rhinitis current, Bet v 1 sensitization (17)	6.30	2.30	17.3	<0.001	6.14	2.09	18.0	0.0011	6.97	2.32	21.0	<0.001
Eczema current, rhinitis current, Cry j 1 sensitization (54)	3.02	1.50	6.08	0.0019	2.68	1.31	5.52	0.0075	4.01	1.86	8.66	<0.001
Wheeze current, eczema current, rhinitis current (13)	7.29	2.36	22.5	<0.001	7.80	2.38	25.6	<0.001	8.26	2.13	32.1	0.0023
Wheeze current, eczema current, rhinitis current, pan-allergen sensitization (12)	6.08	1.86	19.8	0.0028	6.17	1.78	21.3	0.0043	8.26	2.13	32.1	0.0023
Wheeze current, eczema current, rhinitis current, Bet v 1 sensitization (5)	33.9	3.72	308	0.0018	-	-	-	-	35.5	3.55	356	0.0024
Wheeze current, eczema current, rhinitis current, Cry j 1 sensitization (11)	4.77	1.35	16.8	0.015	4.69	1.26	17.4	0.022	6.12	1.48	25.4	0.012

^†^ Multiple imputations; ^‡^ complete data; confounding factors included sex, pet-keeping, smoking exposure, siblings, family income, and parental history of rhinitis. (N) Categorized number; aOR, adjusted odds ratio; CI, confidence interval; OR, odds ratio; PFAS, pollen food allergy syndrome.

## Data Availability

The data presented in this study are available on request from the corresponding author.

## Supplementary Materials

The following supporting information can be downloaded at: https://www.mdpi.com/article/10.3390/nu14132658/s1, Table S1: Definition of exposures and outcomes; Table S2: Pan-allergens for the analysis in this study; Table S3: Comparison of basal characteristics between the included and excluded participants.

## References

[1] Amlot P.L., Kemeny D.M., Zachary C., Parkes P., Lessof M.H. (1987). Oral allergy syndrome (OAS): Symptoms of IgE-mediated hypersensitivity to foods. Clin. Allergy.

[2] Lessof M.H. (1996). Pollen-food allergy syndrome. J. Allergy Clin. Immunol..

[3] Carlson G., Coop C. (2019). Pollen food allergy syndrome (PFAS): A review of current available literature. Ann. Allergy Asthma Immunol..

[4] Matricardi P.M., Kleine-Tebbe J., Hoffmann H.J., Valenta R., Hilger C., Hofmaier S., Aalberse R.C., Agache I., Asero R., Ballmer-Weber B. (2016). EAACI molecular allergology User’s guide. Pediatr. Allergy Immunol..

[5] Geroldinger-Simic M., Zelniker T., Aberer W., Ebner C., Egger C., Greiderer A., Prem N., Lidholm J., Ballmer-Weber B.K., Vieths S. (2011). Birch pollen-related food allergy: Clinical aspects and role of allergen-specific IgE and IgG4 antibodies. J. Allergy Clin. Immunol..

[6] Ciprandi G., Comite P., Ferrero F., Bignardi D., Minale P., Voltolini S., Troisse C., Mussap M. (2016). Birch allergy and oral allergy syndrome: The practical relevance of serum immunoglobulin E to Bet v 1. Allergy Asthma Proc..

[7] Osawa Y., Ito Y., Takahashi N., Sugimoto C., Kohno Y., Mori S., Morikawa T., Kato Y., Okamoto M., Kanno M. (2020). Epidemiological study of oral allergy syndrome in birch pollen dispersal-free regions. Allergol Int..

[8] Kiguchi T., Yamamoto-Hanada K., Saito-Abe M., Sato M., Irahara M., Ogita H., Miyagi Y., Inuzuka Y., Toyokuni K., Nishimura K. (2021). Pollen-food allergy syndrome and component sensitization in adolescents: A Japanese population-based study. PLoS ONE.

[9] Hill D.A., Spergel J.M. (2018). The atopic march: Critical evidence and clinical relevance. Ann. Allergy Asthma Immunol..

[10] Leung D.Y., Guttman-Yassky E. (2014). Deciphering the complexities of atopic dermatitis: Shifting paradigms in treatment approaches. J. Allergy Clin. Immunol..

[11] Paller A.S., Spergel J.M., Mina-Osorio P., Irvine A.D. (2019). The atopic march and atopic multimorbidity: Many trajectories, many pathways. J. Allergy Clin. Immunol..

[12] Tran M.M., Lefebvre D.L., Dharma C., Dai D., Lou W.Y.W., Subbarao P., Becker A.B., Mandhane P.J., Turvey S.E., Sears M.R. (2018). Predicting the atopic march: Results from the Canadian Healthy Infant Longitudinal Development study. J. Allergy Clin. Immunol..

[13] Belgrave D.C., Granell R., Simpson A., Guiver J., Bishop C., Buchan I., Henderson A.J., Custovic A. (2014). Developmental profiles of eczema, wheeze, and rhinitis: Two population-based birth cohort studies. PLoS Med..

[14] Yamamoto-Hanada K., Borres M.P., Åberg M.K., Yang L., Fukuie T., Narita M., Saito H., Ohya Y. (2020). IgE responses to multiple allergen components among school-aged children in a general population birth cohort in Tokyo. World Allergy Organ. J..

[15] Yamamoto-Hanada K., Yang L., Narita M., Saito H., Ohya Y. (2017). Influence of antibiotic use in early childhood on asthma and allergic diseases at age 5. Ann. Allergy Asthma Immunol..

[16] Yang L., Narita M., Yamamoto-Hanada K., Sakamoto N., Saito H., Ohya Y. (2018). Phenotypes of childhood wheeze in Japanese children: A group-based trajectory analysis. Pediatr Allergy Immunol..

[17] Koseki R., Morii W., Noguchi E., Ishikawa M., Yang L., Yamamoto-Hanada K., Narita M., Saito H., Ohya Y. (2019). Effect of filaggrin loss-of-function mutations on atopic dermatitis in young age: A longitudinal birth cohort study. J. Hum. Genet..

[18] Irahara M., Yamamoto-Hanada K., Yang L., Saito-Abe M., Sato M., Inuzuka Y., Toyokuni K., Nishimura K., Ishikawa F., Miyaji Y. (2020). Impact of swimming school attendance in 3-year-old children with wheeze and rhinitis at age 5 years: A prospective birth cohort study in Tokyo. PLoS ONE.

[19] Hamilton R.G., Hemmer W., Nopp A., Kleine-Tebbe J. (2020). Advances in IgE testing for diagnosis of allergic disease. J. Allergy Clin. Immunol. Pract..

[20] Martínez-Aranguren R., Lizaso M.T., Goikoetxea M.J., García B.E., Cabrera-Freitag P., Trellez O., Sanz M.L. (2014). Is the determination of specific IgE against components using ISAC 112 a reproducible technique?. PLoS ONE.

[21] Hauser M., Roulias A., Ferreira F., Egger M. (2010). Panallergens and their impact on the allergic patient. Allergy Asthma Clin. Immunol..

[22] Asher M.I., Keil U., Anderson H.R., Beasley R., Crane J., Martinez F., Mitchell E.A., Pearce N., Sibbald B., Stewart A.W. (1995). International study of asthma and allergies in childhood (ISAAC): Rationale and methods. Eur. Respir. J..

[23] Weiland S.K., Björkstén B., Brunekreef B., Cookson W.O., Von Mutius E., Strachan D.P., International Study of Asthma and Allergies in Childhood Phase II Study Group (2004). Phase II of the international study of asthma and allergies in childhood (ISAAC II): Rationale and methods. Eur. Respir. J..

[24] Ellwood P., Asher M.I., Beasley R., Clayton T.O., Stewart A.W., ISAAC Steering Committee (2005). The international study of asthma and allergies in childhood (ISAAC): Phase three rationale and methods. Int. J. Tuberc. Lung Dis..

[25] Marrs T., Logan K., Craven J., Radulovic S., McLean W.H.A.I., Lack G., Lack G., Flohr C., Perkin M.R., EAT Study Team (2019). Dog ownership at three months of age is associated with protection against food allergy. Allergy.

[26] Gao X., Yin M., Yang P., Li X., Di L., Wang W., Cui H., Yan X., Liu J. (2020). Effect of exposure to cats and dogs on the risk of asthma and allergic rhinitis: A systematic review and meta-analysis. Am. J. Rhinol. Allergy.

[27] Pawankar R., Holgate S.T., Canonica W., Lockey R.F., Blaiss M.S. (2013). White Book on Allergy 2013 Update.

[28] Pawankar R. (2014). Allergic diseases and asthma: A global public health concern and a call to action. World Allergy Organ. J..

[29] Hill D.A., Grundmeier R.W., Ram G., Spergel J.M. (2016). The epidemiologic characteristics of healthcare provider-diagnosed eczema, asthma, allergic rhinitis, and food allergy in children: A retrospective cohort study. BMC Pediatr..

