# Supplementary material for: Pollen Food Allergy Syndrome in Allergic March
Source: Nutrients. 2022 Jun 27;14(13):2658. doi: 10.3390/nu14132658 (PMC9268136; doi:10.3390/nu14132658)
Supplement: Supplementary file 1 [file nutrients-14-02658-s001.zip › nutrients-1760749-supplementary.pdf]

Table S1. Definition of exposures and outcomes

Exposures	Definitions
Wheeze current at 5 years of age	A positive answer from the caregiver to the question (child at 5 y old), “Has your child ever had wheezing or whistling in the past 12 months?”
Wheeze current at 9 years of age	A positive answer from the caregiver to the question (child at 9 y old), “Has your child ever had wheezing or whistling in the past 12 months?”
Eczema current at 5 years of age	A positive answer from the caregiver to the question (child at 5 y old), “Has your child ever had itchy eczema in the past 12 months?”
Eczema current at 9 years of age	A positive answer from the caregiver to the question (child at 9 y old), “Has your child ever had itchy eczema in the past 12 months?”
Rhinitis current at 5 years of age	A positive answer from the caregiver to the question (child at 5 y old),“In the past 12 months, has your child had a problem with sneezing, or a runny, or blocked nose when he/she did not have a cold or the flu?”
Rhinitis current at 9 years of age	A positive answer from the caregiver to the question (child at 9 y old),“In the past 12 months, has your child had a problem with sneezing, or a runny, or blocked nose when he/she did not have a cold or the flu?”
Pan-allergen sensitization status at 5 years of age	Specific IgE ≧ 0.3 ISU for any pan-allergen (child at 5 y old) was considered positive. Specific IgE antibody levels were measured within the range of 0.3–100 ISU-E (ISAC standardized units).
Pan-allergen sensitization status at 9 years of age	Specific IgE ≧ 0.3 ISU for any pan-allergen (child at 9 y old) was considered positive. Specific IgE antibody levels were measured within the range of 0.3–100 ISU-E (ISAC standardized units).

Sensitization to Bet v1 sIgE at 5 years of age	Specific IgE ≧ 0.3 ISU for Bet v1 (child at 5 y old) was considered positive. Specific IgE antibody levels were measured within the range of 0.3–100 ISU-E (ISAC standardized units).
Sensitization to Bet v1 sIgE at 9 years of age	Specific IgE ≧ 0.3 ISU for Bet v1 (child at 9 y old) was considered positive. Specific IgE antibody levels were measured within the range of 0.3–100 ISU-E (ISAC standardized units).
Sensitization to Cry j 1 sIgE at 5 years of age	Specific IgE ≧ 0.3 ISU for Cry j 1 (child at 5 y old) was considered positive. Specific IgE antibody levels were measured within the range of 0.3–100 ISU-E (ISAC standardized units).
Sensitization to Cry j 1 sIgE at 9 years of age	Specific IgE ≧ 0.3 ISU for Bet v1 (child at 9 y old) was considered positive. Specific IgE antibody levels were measured within the range of 0.3–100 ISU-E (ISAC standardized units).
Sensitization to Der p 1 sIgE at 5 years of age	Specific IgE ≧ 0.3 ISU for Der p 1(child at 5 y old) was considered positive. Specific IgE antibody levels were measured within the range of 0.3–100 ISU-E (ISAC standardized units).
Sensitization to Der p 1 sIgE at 9 years of age	Specific IgE ≧ 0.3 ISU for Der p 1 (child at 9 y old) was considered positive. Specific IgE antibody levels were measured within the range of 0.3–100 ISU-E (ISAC standardized units).
Sensitization to Der f 1 sIgE at 5 years of age	Specific IgE ≧ 0.3 ISU for Der f 1(child at 5 y old) was considered positive. Specific IgE antibody levels were measured within the range of 0.3–100 ISU-E (ISAC standardized units).
Sensitization to Der f 1 sIgE at 9 years of age	Specific IgE ≧ 0.3 ISU for Der f 1 (child at 9 y old) was considered positive. Specific IgE antibody levels were measured within the range of 0.3–100 ISU-E (ISAC standardized units).
Sensitization to Can f 1 sIgE at 5 years of age	Specific IgE ≧ 0.3 ISU for Can f 1 (child at 5 y old) was considered positive. Specific IgE antibody levels were measured within the range of 0.3–100 ISU-E (ISAC standardized units).
Sensitization to Can f 1 sIgE at 9 years of age	Specific IgE ≧ 0.3 ISU for Can f 1 (child at 9 y old) was considered positive. Specific IgE antibody levels were measured within the range of 0.3–100 ISU-E (ISAC standardized units).

Sensitization to Fel d 1 sIgE at 5 years of age	Specific IgE ≧ 0.3 ISU for Fel d 1 (child at 5 y old) was considered positive. Specific IgE antibody levels were measured within the range of 0.3–100 ISU-E (ISAC standardized units).
Sensitization to Fel d 1 sIgE at 9 years of age	Specific IgE ≧ 0.3 ISU for Fel d 1 (child at 9 y old) was considered positive. Specific IgE antibody levels were measured within the range of 0.3–100 ISU-E (ISAC standardized units).
Outcomes	Definitions
Hay fever	A positive answer from the caregiver to the question (child at 13y old), "Has your child ever had hay fever?" (Hay fever ever at 13y)
OAS	A positive answer from the caregiver to the question (child at 13y old), "Has your child ever had an itchy mouth or redness around his/her mouth after eating fruits and vegetables?" (OAS ever at 13y)
Pollen allergy	"Hay fever ever at 13y" and "sensitization to pan allergens"
PFAS	"Pollen allergy ever at 13y" and "OAS ever at 13y"

IgE, immunoglobulin E; ISAAC: international study of asthma and allergies in childhood; OAS, Oral allergy syndrome; PFAS, pollen-food allergy syndrome

Table S2. Pan-allergens for the analysis in this study

Allergen	Species	Protein family
Act d 1	Actinidia deliciosa (Kiwi)	Cysteine protease
Act d 2	Actinidia deliciosa (Kiwi)	Thaumatin-like protein
Act d 5	Actinidia deliciosa (Kiwi)	Kiwellin
Act d 8	Actinidia deliciosa (Kiwi)	PR-10
Aln g 1	Alnus glutinosa (Alder)	PR-10
Amb a 1	Ambrosia artemisiifolia (Ragweed)	Pectate lyase
Api g 1	Apium graveolens (Celery)	PR-10
Ara h 8	Arachis hypogaea (Peanut)	PR-10
Art v 1	Artemisia vulgaris (Mugwort)	Defensin
Art v 3	Artemisia vulgaris (Mugwort)	LTP
Bet v 1	Betula verrucosa (Birch)	PR-10
Bet v 2	Betula verrucosa (Birch)	Profilin
Bet v 4	Betula verrucosa (Birch)	Polcalcin
Cor a 1.0101	Corylus avellana (Hazelnut)	PR-10
Cor a 1.0401	Corylus avellana (Hazelnut)	PR-10
Cor a 8	Corylus avellana (Hazelnut)	nLTP
Cry j 1	Cryptomeria japonica (Japanese cedar)	Pectate lyase
Cup a 1	Cupressus arizonica (Cypress)	Pectate lyase
Cyn d 1	Cynodon dactylon (Bermuda grass)	Beta-expansin
Gly m 4	Glycine max (Soybean)	PR-10
Mal d 1	Malus domestica (Apple)	PR-10
Mer a 1	Mercurialis annua (Annual mercury)	Profilin
Ole e 1	Olea europaea (Olive)	Olive Group 1
Par j 2	Parietaria judaica (Pellitory of the wall)	nLTP
Phl p1	Phleum pratense (Timothy)	Beta-expansin
Phl p 2	Phleum pratense (Timothy)	Grass group II
Phl p 4	Phleum pratense (Timothy)	Berberine bridge enzyme
Phl p 5	Phleum pratense (Timothy)	Grass group V
Phl p 6	Phleum pratense (Timothy)	Grass group VI
Phl p 7	Phleum pratense (Timothy)	Polcalcin

Phl p 11	Phleum pratense (Timothy)	Ole e 1-related protein
Phl p 12	Phleum pratense (Timothy)	Profilin
Pla a 1	Platanus acerifolia (Plane)	Invertase inhibitor
Pru p 1	Prunus persica (Peach)	PR-10
Pru p 3	Prunus persica (Peach)	nLTP
Sal k 1	Salsola komarovii (Saltwort)	Pectin methylesterase

Table S3. Comparison of basal characteristics between the included and excluded participants

	Data included in the analysis			Data not included in the analysis		
Variables	Category	N	%	N	%	P
Sex	male	259	49.7	551	53.5	0.169
	female	262	50.3	478	46.5	
	missing	0		0		
Wheeze current at 5 years of age	no	414	80.9	582	85.3	**0.048**
	yes	98	19.1	100	14.7	
	missing	9		347		
Eczema current at 5 years of age	no	384	75	507	74.4	0.881
	yes	128	25	174	25.6	
	missing	9		348		
Rhinitis current at 5 years of age	no	314	61.3	460	67.4	**0.033**
	yes	198	38.7	222	32.6	
	missing	9		347		
Wheeze current at 9 years of age	no	461	89.2	362	92.8	0.078
	yes	56	10.8	28	7.2	
	missing	4		639		
Eczema current at 9 years of age	no	409	79.1	314	80.7	0.607
	yes	108	20.9	75	19.3	
	missing	4		640		
Rhinitis current at 9 years of age	no	226	43.7	189	48.5	0.175
	yes	291	56.3	201	51.5	
	missing	4		639		
Pet keeping at 6 months	no	419	81.5	674	76.5	**0.034**
	yes	95	18.5	207	23.5	
	missing	7		148		
Smoking exposure at 5 years of age	no	379	74.3	477	70.1	0.129
	yes	131	25.7	203	29.9	
	missing	11		349		
Siblings	no	316	61	437	51.4	**0.001**
	yes	202	39	413	48.6	
	missing	3		179		
Maternal history of allergy	no	185	36.9	417	42.4	**0.049**
	yes	316	63.1	567	57.6	
	missing	20		45		
Paternal history of allergy	no	250	50.1	531	55	0.083
	yes	249	49.9	434	45	
	missing	22		64		
Maternal history of rhinitis	no	241	48.5	552	56.2	**0.006**
	yes	256	51.5	430	43.8	
	missing	24		47		
Paternal history of	no	307	61.8	630	65.3	0.204

rhinitis	yes	190	38.2	335	34.7	
	missing	24		64		
Income < 4,000,000 yen/year	no	435	93.3	880	92.7	0.752
	yes	31	6.7	69	7.3	
	missing	55		80		
